# Long Non-coding RNAs Contribute to the Inhibition of Proliferation and EMT by Pterostilbene in Human Breast Cancer

**DOI:** 10.3389/fonc.2018.00629

**Published:** 2018-12-18

**Authors:** Yongye Huang, Juan Du, Yan Mi, Tianye Li, Ying Gong, Hongsheng Ouyang, Yue Hou

**Affiliations:** ^1^College of Life and Health Sciences, Northeastern University, Shenyang, China; ^2^Jilin Provincial Key Laboratory of Animal Embryo Engineering, College of Animal Sciences, Jilin University, Changchun, China

**Keywords:** pterostilbene, lncRNAs, cancer, autophagy, epithelial-to-mesenchymal transition

## Abstract

**Background:** There is increasing evidence that long non-coding RNAs (lncRNAs) are involved in the process of carcinogenesis and treatment using chemotherapy. Pterostilbene, a phytochemical agent with natural antioxidant and anti-inflammatory properties, has been shown to modulate oncogenic processes in many cancers. However, there has been limited research on the association between pterostilbene and the expression of lncRNAs.

**Methods:** MCF7 breast cancer cells were treated with various concentrations of pterostilbene and their gene expression profile was analyzed by quantitative real-time PCR, Western blotting and immunofluorescence.

**Results:** Treatment with pterostilbene inhibited cell proliferation and epithelial-to-mesenchymal transition (EMT), and increased cell apoptosis, autophagy and ER stress. The Akt/mTOR pathway was downregulated, but p38 MAPK/Erk signaling was activated in cells following treatment with pterostilbene. Pterostilbene increased the expression of the lncRNAs MEG3, TUG1, H19, and DICER1-AS1 whereas the expression of LINC01121, PTTG3P, and HOTAIR declined. Knockdown of lncRNA H19 resulted in a reduction of the cell invasion, with the cells becoming more sensitive to pterostilbene therapy.

**Conclusions:** These results suggest that efficient optimum disruption of lncRNA expression might possibly improve the anti-tumor effects of phytochemical agents, thus serving as a potential therapy for breast cancer.

## Introduction

Cancer is among the leading causes of death. Distant tumor metastasis is generally considered a major cause of poor survival. Epithelial-to-mesenchymal transition (EMT) is recognized as an important process associated with increased aggressive, invasive and metastatic potential in many types of cancer cells. Furthermore, EMT has been shown to be predictive of tumor response following neoadjuvant chemotherapy (1). There is increasing evidence to suggest that targeting EMT could overcome resistance to chemotherapy.

Many studies have been undertaken to understand the progression from normal cells to tumors and to uncover clinically important tumor-targeting drugs as cancer therapies. However, a number of therapeutic strategies such as these have ironically resulted in increased mortality due to drug resistance and metastatic recurrence. Therefore, more attention has been paid to the development of medicinal and functional dietary herbs or phytochemicals as alternatives to medicines to fight cancer (2, 3). Phytochemical agents have often been utilized as alternative therapies to improve the outcomes of traditional cancer treatment. Pterostilbene is a well-known natural antioxidant extracted from grapes, blueberries and peanuts, and is considered to be one of the more powerful stilbene compounds. It has a structural similarity to resveratrol but exhibits superior pharmacokinetic characteristics. Pterostilbene is reported to have therapeutic potential for many diseases, including cancer, dyslipidemia, aging and inflammatory disorders (4, 5). As an emerging tumor suppressor, pterostilbene is effective in modulating various cancers, including breast cancers (6), by inhibiting the proliferation of breast cancer cells and reducing the expression of human telomerase reverse transcriptase (7). Thus, opposing cancer has been suggested as among the major biological properties of pterostilbene.

Pterostibene has been shown to induce cell cycle arrest, apoptosis, necrosis, and autophagy as effects that resist cancer in many cancer cell lines. For example, in hepatocellular carcinoma, pterostilbene triggers apoptosis by the effective regulation of MTA1/HDAC1/NuRD complex and induction of PTEN acetylation (8). Pterostilbene has been shown to cause a marked increase in the proportion of bladder cancer cells in G0/G1 of the cell cycle in addition to apoptosis and autophagy (9). Endoplasmic reticulum (ER) stress is considered the result of disturbances in homeostasis in the ER, leading to unfolded and misfolded proteins accumulating in the lumens of the ER. Many studies have documented the relationship between ER stress, apoptosis and autophagy, although they function independently. The importance of ER stress-induced apoptosis can be evidenced by the number of human diseases in which anti-apoptotic or pro-apoptotic genes such as Bax or Bcl-2 are activated (10, 11). One study demonstrated that pterostilbene was able to induce apoptosis via activation of the ER stress marker gene CHOP in TRAIL-resistant triple negative breast cancer (TNBC) cells (12). In addition, ER stress and autophagy share numerous characteristics including protection of tissues or organs by the release of stress and the triggering of cell death in response to extreme conditions (13). Anti-cancer activity in pterostilbene has been shown to be result of the elevation of ER stress and inhibition of autophagy in HT1080 cells (14). Thus, we speculate that apoptosis, autophagy and ER stress jointly contribute to the effects against cancer observed following pterostilbene treatment.

Long non-coding RNA (lncRNA) molecules are longer than 200 nucleotides (nt) in length with exceptionally limited protein-coding potential (15). Numerous reports have demonstrated key roles of lncRNAs in the development and progression of breast cancer. Proliferation, apoptosis, autophagy, invasion, and metastasis are all regulated through the expression of lncRNAs (16, 17). For example, increased expression of the lncRNA HOTAIR has been observed in primary breast tumors as well as metastases and, conversely, a decrease in its expression has been shown to prevent cancer invasiveness (18). The lncRNA H19 mediates EMT and the reverse process, mesenchymal-to-epithelial transition (MET), in breast cancer, although it has a role as a differential sponge for the microRNAs miR-200b/c and let-7b (19). The lncRNA MALAT1 regulates cell proliferation and apoptosis via interacting with DBC1 to modulate p53 acetylation (20). LncRNAs have multiple functions in a wide range of biological processes, but little is known about the biological roles that lncRNAs perform in combatting tumors during treatment with pterostilbene.

Survival rates for women with breast cancer have continued to improve significantly over recent decades (21). However, as indicated by the global cancer statistics 2018, breast cancer ranked second out of the most diagnosed cancers, with 11.6% of all cases, even when considering both sexes (22). Considerable work remains in order to find the most efficacious treatments for breast cancers. Therefore, the present study aimed to ascertain additional mechanisms that pterostilbene exhibits in breast cancer cells, especially in its involvement in lncRNA expression.

## Materials and Methods

### Cell Lines and Culture Condition

Human MCF7 cells were cultured in DMEM (Gibco, Carlsbad, CA, USA) containing 10% fetal bovine serum, 1% non-essential amino acid, 1% glutamine, 100 U/mL penicillin, and 100 mg/mL streptomycin in humidified air with 5% CO_2_ at 37°C.

### Drugs

Pterostilbene (purity ≥ 99%) was purchased from Chengdu Pufeide Biotechnology Co., Ltd. A stock solution of pterostilbene was prepared in DMSO and stored at −20°C, then diluted in culture media immediately prior to experimentation. FBS-free DMEM with the same volume of DMSO was used as the control.

### Cell Proliferation Assay

An MTT assay was performed to investigate the effects of pterostilbene treatment on cell proliferation. Four thousand cells were seeded in 96-well plates and exposed to various concentrations of pterostilbene for 6, 12, 24, 36, and 48 h at 37°C in an atmosphere of 5% CO_2_ in a humidified incubator. MTT solution was then added to each well and incubated for an additional 4 h. DMSO was then added and the optical density (OD) at 490 nm measured using a microplate reader. Cell proliferation was expressed as the OD value ± SEM.

### Annexin V-FITC/PI Assay

Cellular apoptosis was evaluated using an AnnexinV/PI double staining assay. Annexin V-FITC stains the membranes of early-stage apoptotic cells (green) and PI stains the nuclei of late apoptotic or necrotic cells (red). After treatment with pterostilbene, cells were washed with PBS, incubated in 100 μL binding buffer containing Annexin V-FITC and propidium iodide (PI) at room temperature for 15 min in the dark. A total of 400 μL binding buffer was added and the cells were then analyzed using a FACScan flow cytometer (Becton-Dickinson, San Jose, USA).

### Quantitative Real-Time PCR

Total RNA was isolated from the cancer cells using TRIzol as described in the manufacturer's manual. The RNA was reverse transcribed to cDNA using All-in-One cDNA synthesis SuperMix (Bimake, Houston, TX, USA). Real-time gene expression was analyzed using a 2x SYBR Green qPCR Master Mix (Bimake, Houston, TX, USA) in a Real-time PCR thermal cycler. Reaction conditions were as follows: 95°C, 15 min; then 45 cycles of 95°C, 10 min; 60°C, 30 min; 72°C, 20 min. Gene expression was quantified after normalization to GAPDH levels and expressed as fold change. All assays were performed in triplicate.

### Protein Extraction and Western Blotting

Cells that were either untreated or treated with pterostilbene were washed in ice-cold PBS three times and lysed in a 300 mL cell lysis buffer supplemented with a cocktail of 1 mM phenylmethanesulfonyl fluoride (PMSF) and protease inhibitors. After incubation for 30 min, the cell lysate was centrifuged and total protein was harvested from the supernatant. The concentration was quantified using a bicinchoninic acid (BCA) protein assay kit (Beyotime, Hangzhou, China). Lysates were separated using an SDS-PAGE gel, transferred to PVDF membranes and then subjected to Western blot analysis. The PVDF membranes were incubated with primary antibodies overnight at 4°C, followed by the appropriate peroxidase-conjugated secondary antibody at room temperature for 2 h. The membranes were washed three times with PBS and blot imaging using an enhanced chemiluminescent (ECL) detection system.

### Immunofluorescent Detection

Cells were also cultured on coverslips for additional analysis. After rinsing briefly with PBS, the cells were fixed in 4% paraformaldehyde in PBS for 30 min. They were permeabilized using 0.2% Triton X-100 in PBS for 30 min on ice, then subsequently washed with PBS three times. Non-specific binding was blocked by adding goat serum for 30 min at room temperature and then appropriate primary antibodies were added at dilutions indicated by the manufacturer; they were then incubated overnight at 4°C. The primary antibodies were removed and the cells were washed vigorously with 0.2% Tween-20 in PBS. They were then incubated with the appropriate secondary antibodies overnight at 4°C. After washing with PBS, the cells were stained with Hoechst 33,342 for 5 min at 37°C, fluorescent images acquired using an Olympus FV1000 confocal microscope (Olympus, Japan).

### Migration Assay

Boyden chamber migration assays were performed using 24-well Transwell chambers with 8-μm polycarbonate nucleopore filters. Briefly, cells were either untreated or treated with pterostilbene, trypsinized then added to the upper Transwell chamber at a density of 5 × 10^4^ cells/well in 200 μL serum-free DMEM with 600 μL DMEM containing 2.5% FBS added to each lower chamber. After incubating for 24 h at 37°C, cells that had migrated and adhered to the lower surface of the membrane were fixed with methanol for 30 min at room temperature and then stained with 0.1% crystal violet for 20 min. Cells were enumerated using a light microscope.

### Statistical Analysis

Statistical analyses were performed using SPSS software (Chicago, IL, USA). Data were considered statistically significant when *p* < 0.05. Each experiment was conducted in triplicate and repeated three times. Results were expressed as mean ± SEM.

## Results

### Pterostilbene Inhibited Cell Proliferation and Was Accompanied by a Change in Caspase-3 and Caspase-9 Expression.

**Figure 1:**
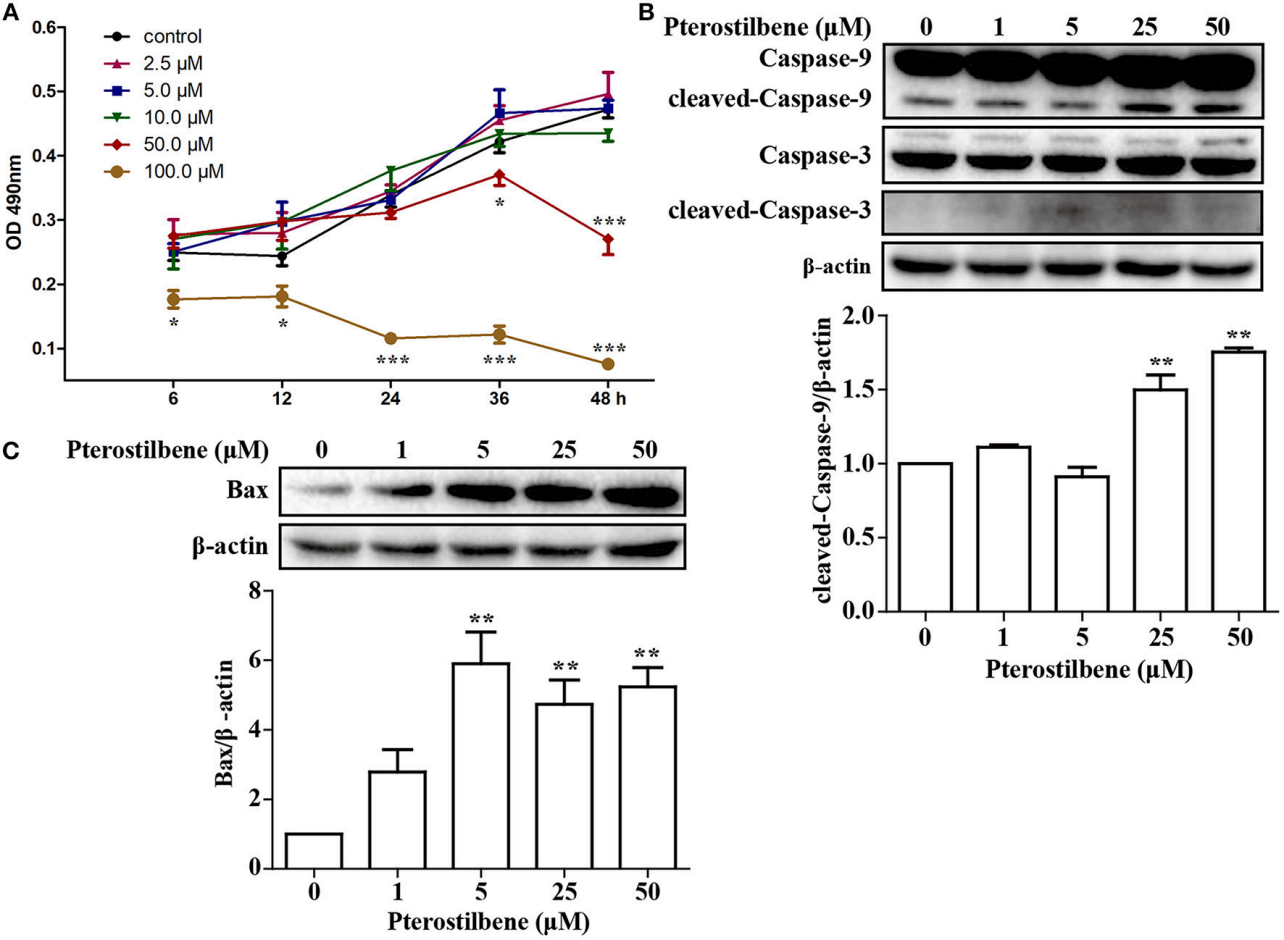
Effect of pterostilbene on the viability of cancer cells: **(A)** Cell proliferation at different treatment concentrations and time points; **(B)** Expression of caspase-3 and−9 as determined by Western blot analysis; **(C)** Expression of Bax as evaluated by Western blot analysis. Reported values are mean ± SEM. *p < 0.05, **p < 0.01 and ***p < 0.001, indicate significant differences compared with the control group.

An MTT assay was performed to ascertain the effects of pterostilbene on the proliferation of MCF7 breast cancer cells. The cells were treated with various concentrations of pterostilbene (0, 2.5, 5, 10, 50, and 100 μM) over different durations (6, 12, 24, 36, and 48 h). The results revealed that the proliferation of cells treated with 100 μM pterostilbene was significantly inhibited at the start of the treatment (Figure 1A), whereas inhibition with 50 μM pterostilbene was significant at 24 and 48 h. These results suggest that pterostilbene inhibited cell proliferation in a dose- and time-dependent manner. The IC_50_ values in MCF7 cells were 175.62, 83.09 and 53.21 μM after 6, 24 and 48 h incubation, respectively (Table 1).

**Table 1:** IC_50_ value of pterostilbene inhibition of cell viability.

**Time (h)**	**IC_**50**_ value (μM)**
6	175.62 ± 0.93
24	83.09 ± 0.36
48	53.20 ± 0.79

**Figure 2:**
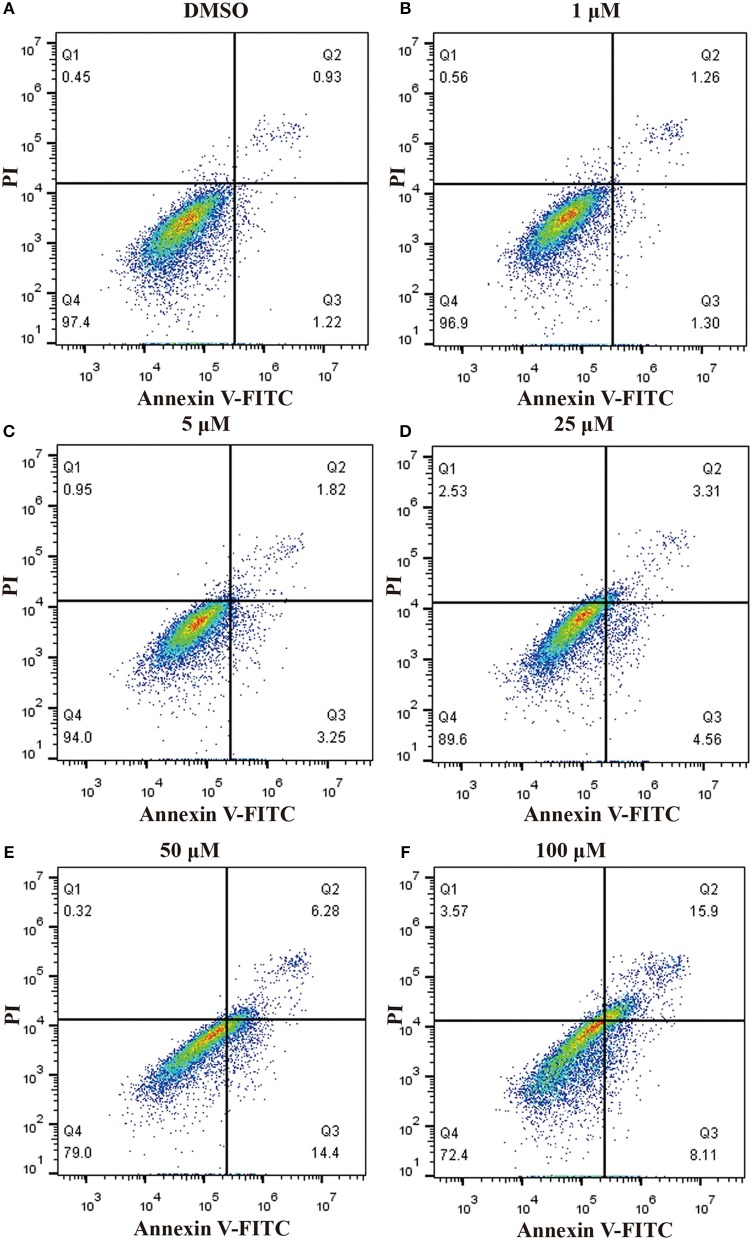
Pterostilbene induced apoptosis in MCF7 cancer cells s. Apoptosis in cells untreated and treated with pterostilbene (**A**, control; **B**, 1 μM; **C**, 5 μM; **D**, 25 μM; **E**, 50 μM; **F**, 100 μM) was evaluated by flow cytometry using Annexin V-FITC/PI staining.

In order to ascertain whether inhibition of proliferation induced by pterostilbene was associated with increased activation of the apoptotic pathway, the level of apoptosis in pterostilbene-treated cells was analyzed using flow cytometry. Pterostilbene induced apoptosis in a dose dependent manner (Figure 2) and, specially, doses of 50 and 100 μM induced apoptosis in >20% of cells. The expression of genes involved in cell survival following pterostilbene treatment was also evaluated. Treatment increased the cleavage of caspase-3 and caspase-9, two molecular markers of apoptosis (Figure 1B), and the expression of Bax, a pro-apoptotic protein, in a dose dependent manner (Figure 1C). These results suggest that pterostilbene treatment activated the caspase-dependent apoptotic pathway.

### Formation of LC3-II Protein Was Induced by Pterostilbene Treatment

**Figure 3:**
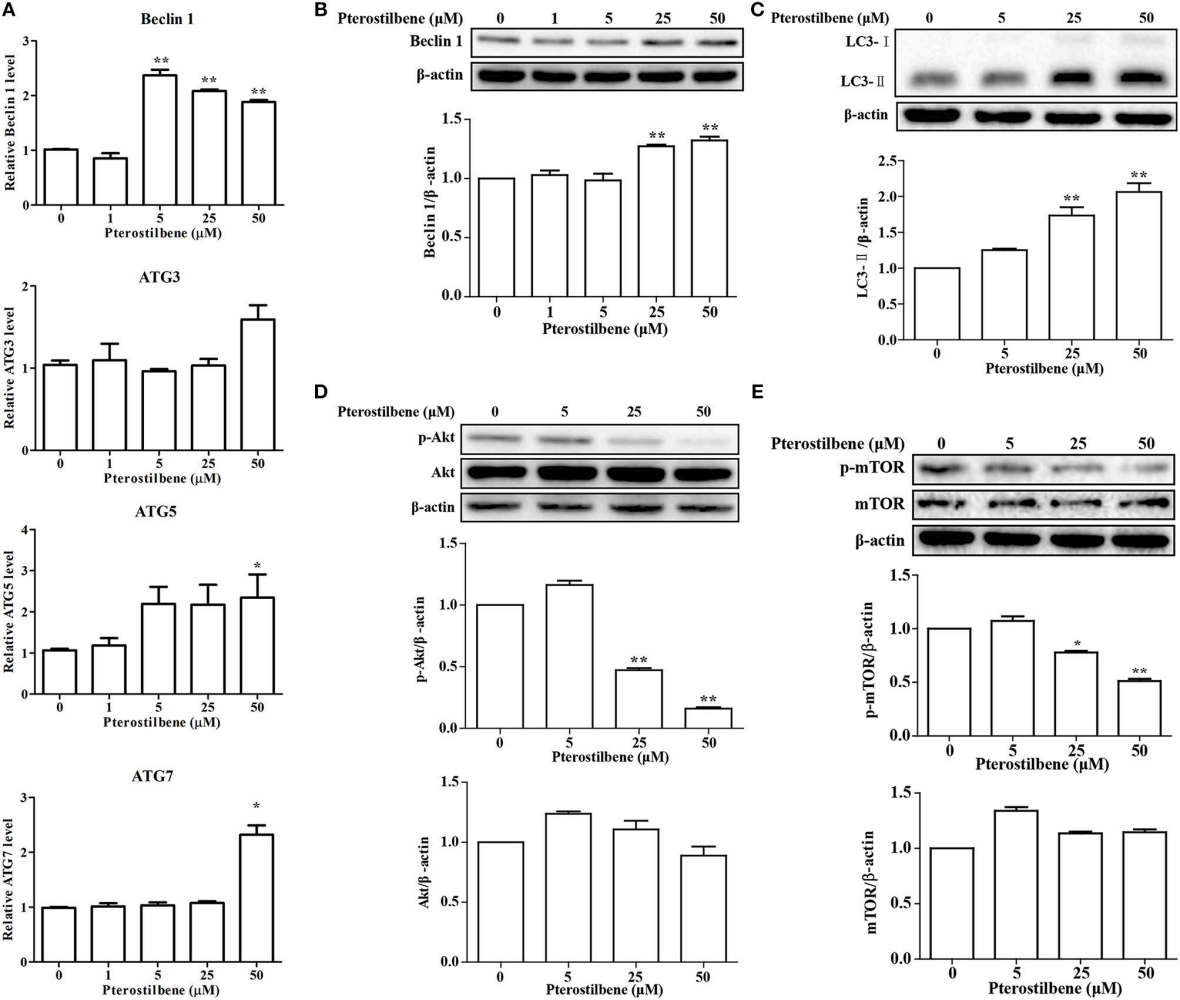
Pterostilbene triggered autophagy in cancer cells: **(A)** Expression of autophagy-related genes determined by qRT-PCR; **(B–E)** Expression of autophagy-related genes by Western blot analysis. Reported values are mean ± SEM. *p < 0.05 and **p < 0.01, indicate significant differences compared with the control group.

Emerging evidence has revealed the importance of autophagy in the regulation of tumor development. Autophagic cell death was examined after treatment with pterostilbene to explore whether it can trigger autophagy by measuring the expression of the autophagosome marker Beclin 1. The qRT-PCR results indicated that mRNA expression levels of Beclin 1 increased significantly at pterostilbene concentrations of 5, 25, and 50 μM, as shown in Figure 3A. Western blot analysis confirmed that the expression of Beclin 1 protein increased in a dose-dependent manner, with significant increase observed at 25 and 50 μM (Figure 3B). It is known that a cytosolic form of LC3 (LC3-I) conjugates to phosphatidylethanolamine to form membrane-bound LC3 (LC3-II) during autophagy, the formation of which was found to increase after pterostilbene treatment in a dose-dependent manner (Figure 3C). ATG3, ATG5, and ATG7 mRNA levels were also quantified by qRT-PCR. The results demonstrate that ATG5 and ATG7 expression were enhanced after pterostilbene treatment at 50 μM for 24 h (Figure 3A). Given that the Akt-mTOR pathway is key in the regulation of autophagy, we measured the phosphorylation of Akt-mTOR by Western blotting. As shown in Figure 3D, treatment with pterostilbene for 24 h reduced the phosphorylation of Akt protein. In addition, exposure of cancer cells to pterostilbene resulted in a decrease in the phosphorylated (activated) form of mTOR (Ser2448) (Figure 3E). To summarize, these results indicate that pterostilbene treatment triggered Beclin-1-independent autophagy via promotion of LC3-II formation prior to inactivation of the Akt-mTOR pathway.

### Pterostilbene Induced Expression of ER Stress-Related Genes

**Figure 4:**
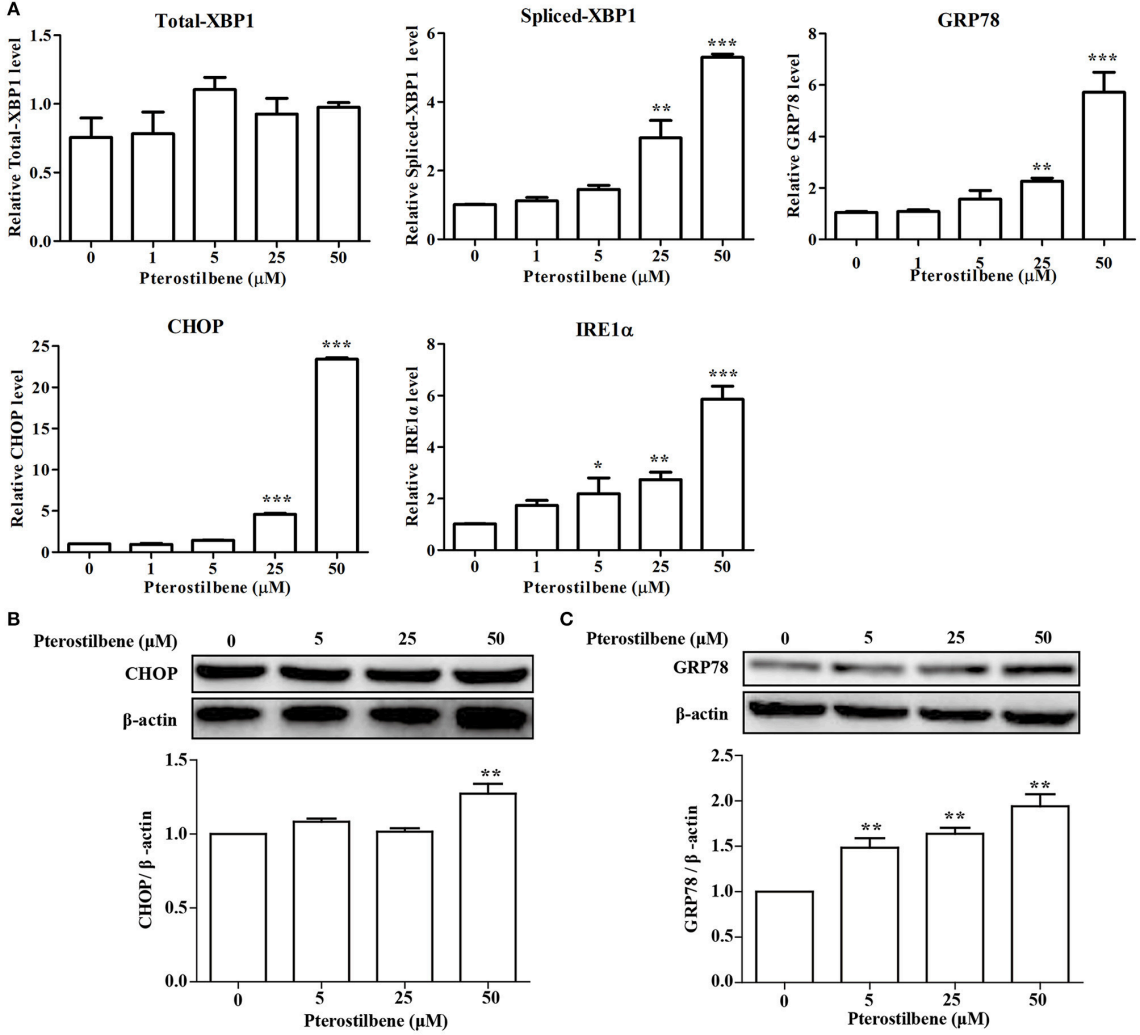
Effect of pterostilbene on the expression of ER stress-related genes. **(A)** mRNA expression of ER stress-related genes; **(B,C)** Expression of autophagy-related genes at the protein level. Reported values are mean ± SEM. *p < 0.05, **p < 0.01 and ***p < 0.001, indicate significant differences compared with the control group.

There is emerging evidence that ER stress may be a cause of apoptosis and autophagy (23, 24) and so the expression of ER stress-associated genes following pterostilbene treatment was examined to evaluate the role of ER stress in the antitumor effects of pterostilbene. The qRT-PCR results indicated that an increase in XBP1 splicing was observed when treated with 50 μM pterostilbene for 24 h (Figure 4A). Furthermore, to further verify the results, additional ER stress marker genes were also studied. As shown in Figure 4A, the expression of GRP78, CHOP and IRE1α increased in a dose-dependent manner relative to pterostilbene. The Western blotting results also confirmed that the expression of GRP78 steadily increased as pterostilbene treatment increased from 5 to 50 μM, and the expression of CHOP was significantly upregulated with a treatment of 50 μM pterostilbene (Figures 4B,C). Together, these findings indicate that ER stress contributes to the anti-tumor effects of pterostilbene.

### Expression of EMT-Associated Genes Was Reversed With Pterostilbene Treatment

**Figure 5:**
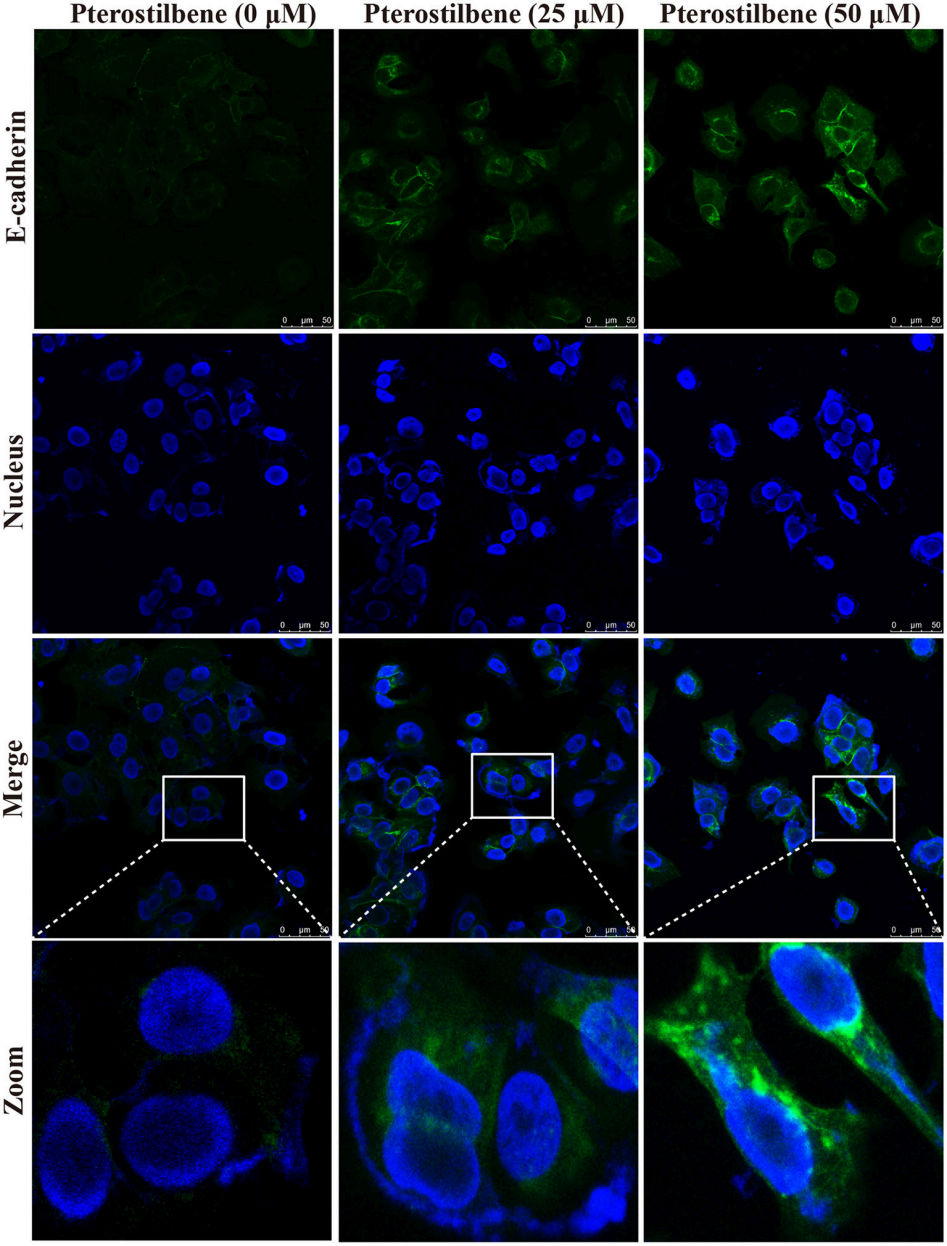
Pterostilbene influenced the expression of E-cadherin. Cells were treated with different concentrations of pterostilbene for 24 h with immunofluorescence observed using confocal fluorescence microscopy.

**Figure 6:**
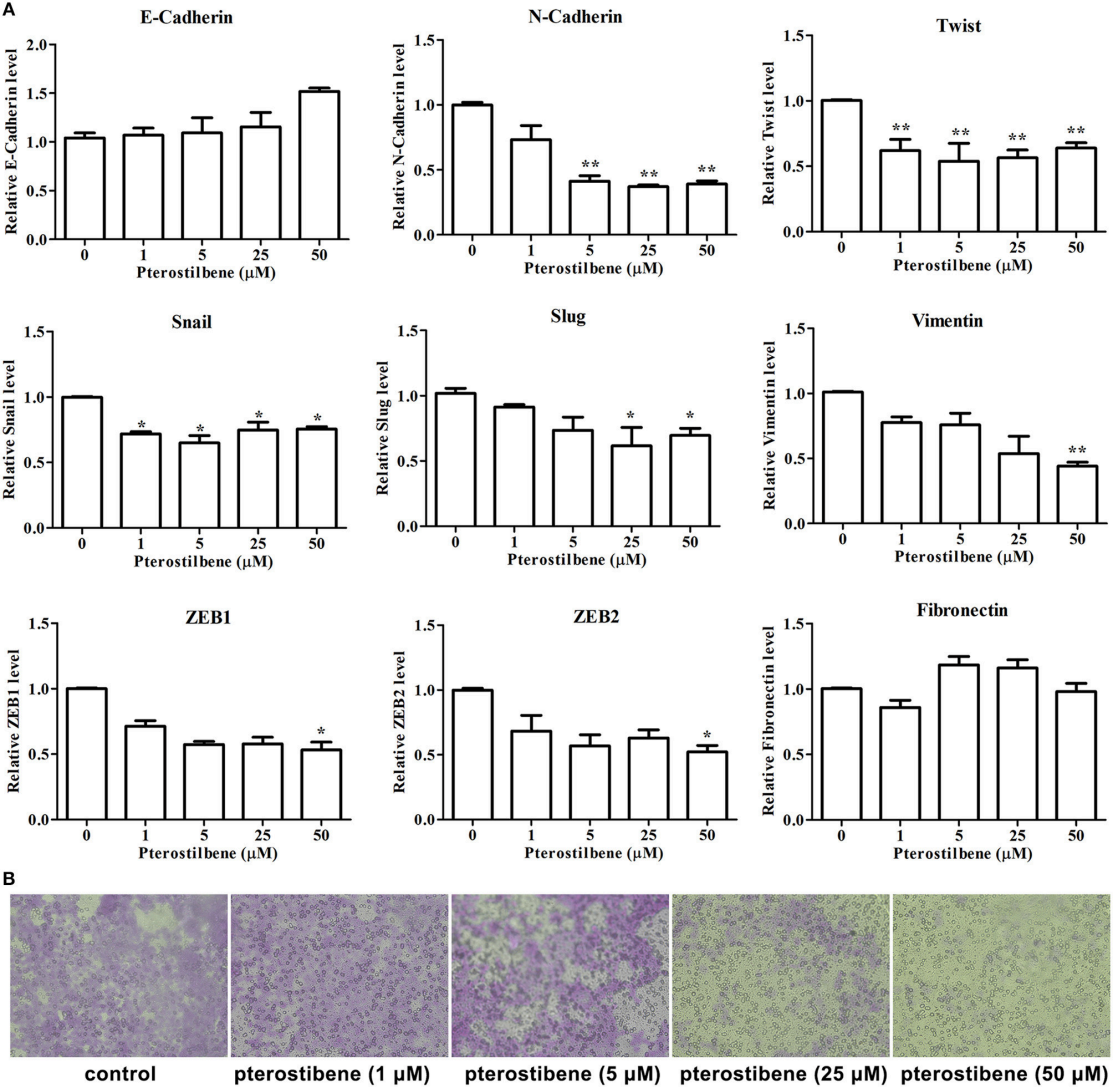
Pterostilbene reduced EMT and migration. **(A)** Pterostilbene regulated the expression of EMT-related genes. Gene expression in cells treated with pterostilbene at different concentrations for 24 h was calculated using qRT-PCR. Reported values are mean ± SEM. *p < 0.05 and **p < 0.01, indicate significant differences compared with the control group. **(B)** Cell migration examined by Transwell assay.

**Figure 7:**
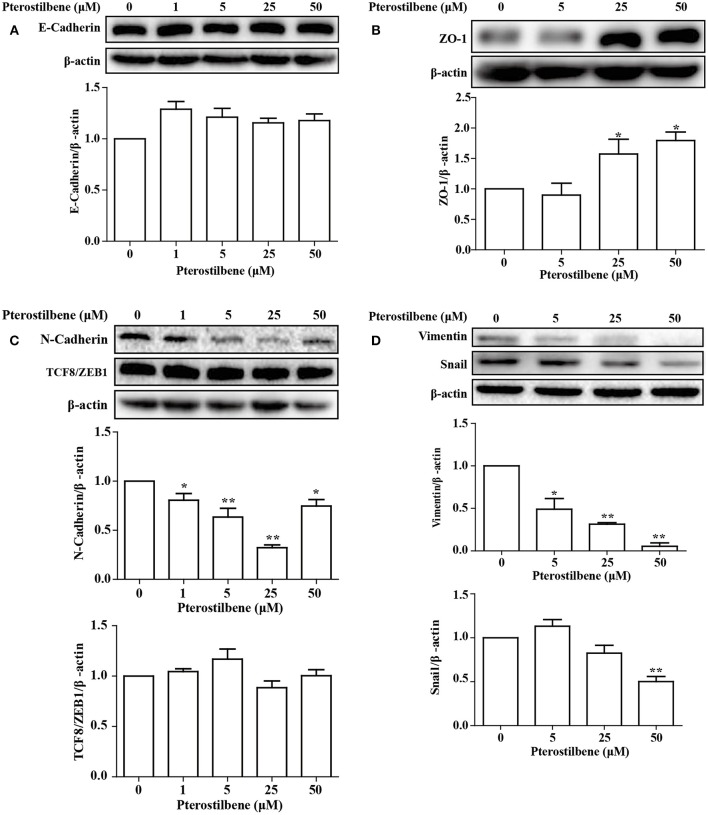
Pterostilbene influenced expression of EMT-related genes (**A**, E-cadherin; **B**, ZO-1; **C**, N-cadherin, and TCF8/ZEB1; **D**, Vimentin, and Snail). Gene expression in cells treated with pterostilbene at different concentrations for 24 h was quantified by Western blot analysis. Reported values are mean ± SEM. *p < 0.05 and **p < 0.01, indicate significant differences compared with the control group.

**Figure 8:**
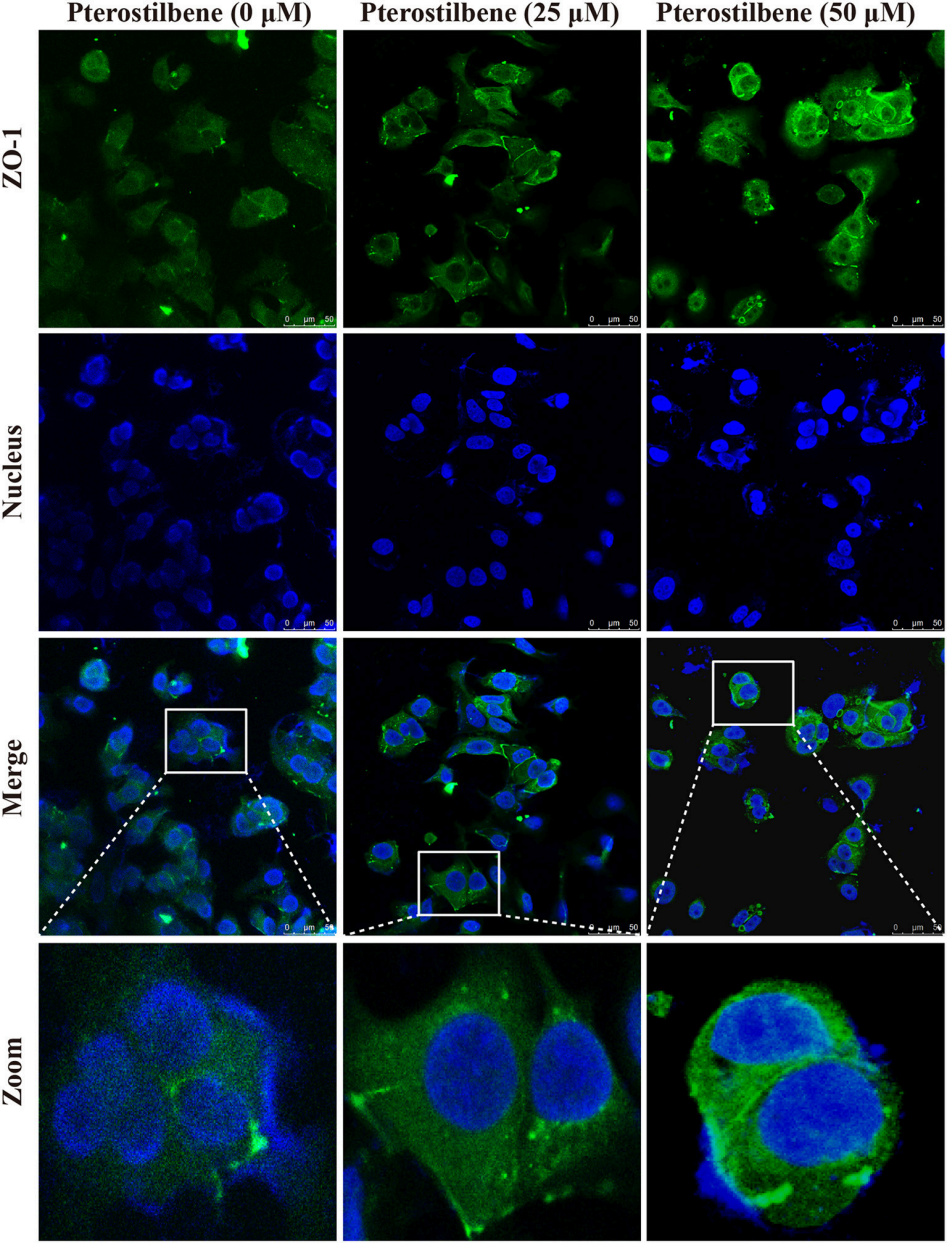
Immunofluorescent staining of ZO-1 expression. Cells were treated with pterostilbene at different concentrations for 24 h, images captured using confocal fluorescence microscopy.

The EMT process contributes to the formation of cancer stem-like characteristics and chemoresistance. To ascertain the effect of pterostilbene on the EMT process, relevant markers and related transcription factors were measured. Compared with the control, increased E-cadherin immunofluorescent staining was observed in the pterostilbene-treated cells (Figure 5). However, the qRT-PCR results indicated that E-cadherin expression was only slightly upregulated after treatment of the MCF7 cells with 50 μM pterostilbene (Figure 6A). No significant difference in the protein expression of E-cadherin was observed among the different treatment concentrations, although a small increase in the relative IOD values was observed in the treated groups (Figure 7A). In order to further confirm the influence of pterostilbene treatment on the expression of epithelial cell marker genes, the expression of ZO-1 was evaluated. As suggested by Western blot analysis, the expression of ZO-1 was upregulated in MCF7 cells after treatment with 25 and 50 μM pterostilbene (Figure 7B). The immunofluorescence staining also showed that ZO-1 was over-expressed after pterostilbene treatment, especially the 50 μM treatment (Figure 8). The above findings demonstrate that the expression of epithelial cell marker genes was increased after pterostilbene treatment.

**Figure 9:**
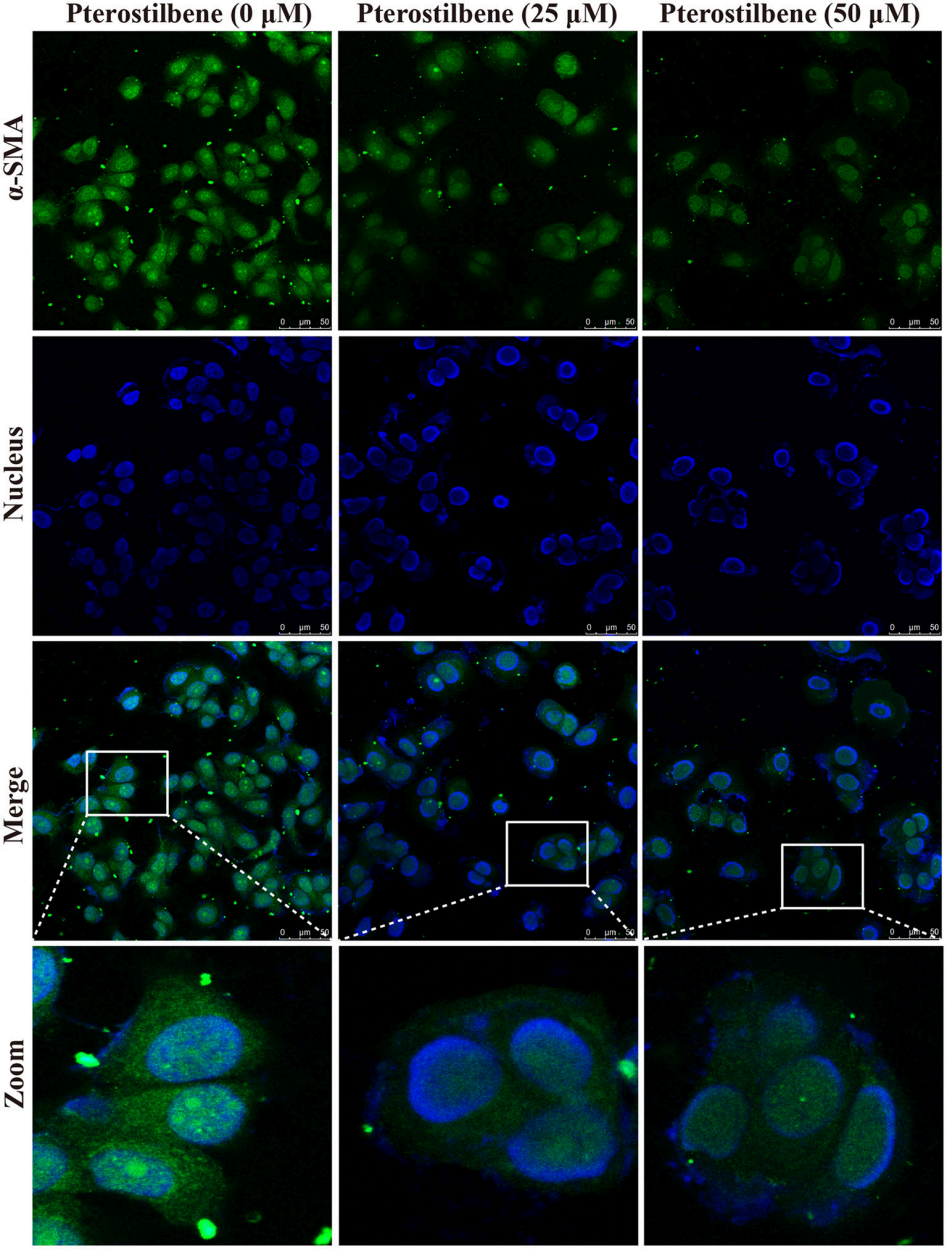
Pterostilbene inhibited α-SMA expression as revealed by immunofluorescent staining. Cells were treated with pterostilbene at different concentrations for 24 h, images captured using confocal fluorescence microscopy.

The expression of the mesenchymal cell marker genes N-cadherin, Zeb1, Zeb2, Vimentin, Snail, Slug, Twist, and fibronectin were subsequently analyzed (Figure 6A). As demonstrated by qRT-PCR, the expression of N-cadherin was downregulated in MCF7 cells after 5, 25, and 50 μM pterostilbene treatment. The mRNA expression of Twist and Snail was downregulated in pterostilbene-treated cells over a range of 1 to 50 μM. Decreased mRNA expression of Vimentin, Slug, Zeb1, and Zeb2 was observed at high dose treatments. However, there was no significant difference in the mRNA expression of fibronectin among the tested groups. To confirm the above results, the gene expression results were compared with Western blot analysis. The expression of N-cadherin at the protein level was consistent with mRNA expression results, but the protein expression of Zeb1 was similar to both the control and the pterostilbene-treated cells (Figure 7C). Protein expression of vimentin was downregulated in cells treated with 5, 25, and 50 μM pterostilbene, and Snail protein expression decreased with the 50 μM pterostilbene treatment (Figure 7D). Immunofluorescence staining was performed to detect the expression of the mesenchymal cell marker α-SMA, the results indicating that it was inhibited when treated with 25 and 50 μM pterostilbene (Figure 9). These results indicate that the expression of mostly EMT-associated genes was disrupted after pterostilbene treatment.

To further validate the effect of pterostilbene treatment on the process of EMT, the capability of cells to migrate was evaluated using a Transwell assay. The results indicate that there was no significant difference between the control and 1 μM pterostilbene-treated cells (Figure 6B), but the rate of migration decreased at a concentration of 5 μM and dramatically so at 25 and 50 μM pterostilbene, compared with control cells.

### Pterostilbene Induced Activation of the MAPK Signaling Pathway

**Figure 10:**
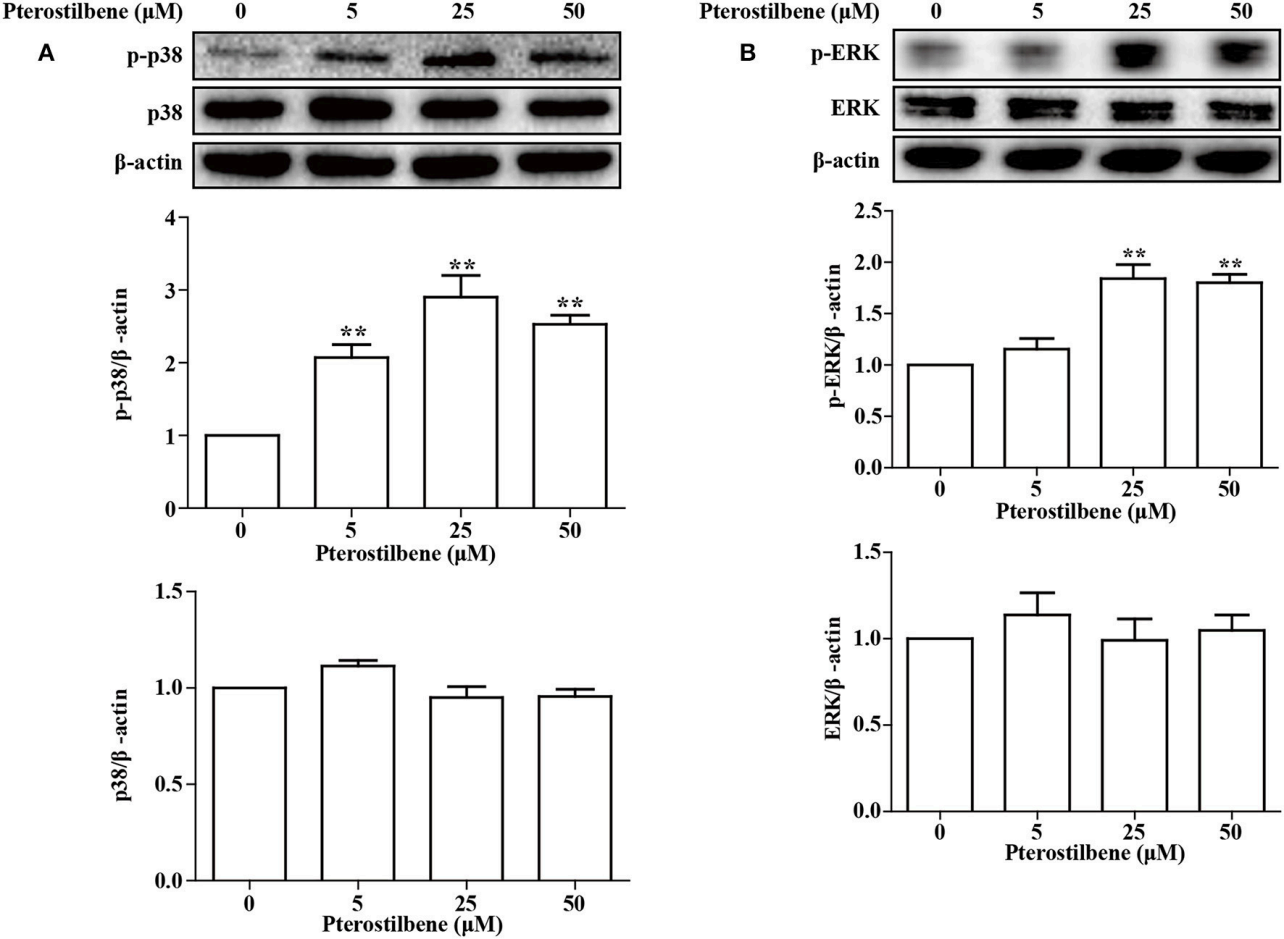
Influence of pterostilbene on p38 and ERK1/2 MAPK pathways. Cells were untreated or treated with pterostilbene (0, 5, 25, and 50 μM) for 24 h and analyzed by Western blotting. Reported values are mean ± SEM. **p < 0.01 indicate significant differences compared with the control group.

The MAPKs (Erk, Jun and p38) have been demonstrated to be involved in EMT. Besides being required for EMT (25), p38 MAPK also functions by controlling the balance of chemotherapeutic agent-induced apoptosis and autophagy (26). Therefore, the p38 MAPK pathway was analyzed in the present study. As shown in Figure 10, little difference was observed in the expression of p38 MAPK between the control and treatment groups, and exposure of MCF7 cells to pterostilbene resulted in increased levels of phosphorylated (activated) p38 MAPK. The Erk1/2 signaling pathway also contributes to the progression and metastasis of breast cancer, and thus resistance to treatment. The results of Western blot analysis demonstrated that treatment with 25 and 50 μM pterostilbene enhanced the level of phosphorylated Erk (Thr202/Tyr204). Therefore, these data indicated that both p38 and Erk1/2 pathways may be involved in the anti-cancer properties exhibited by pterostilbene.

### Expression Profile of lncRNAs in Cancer Cells Following Pterostilbene Treatment

**Figure 11:**
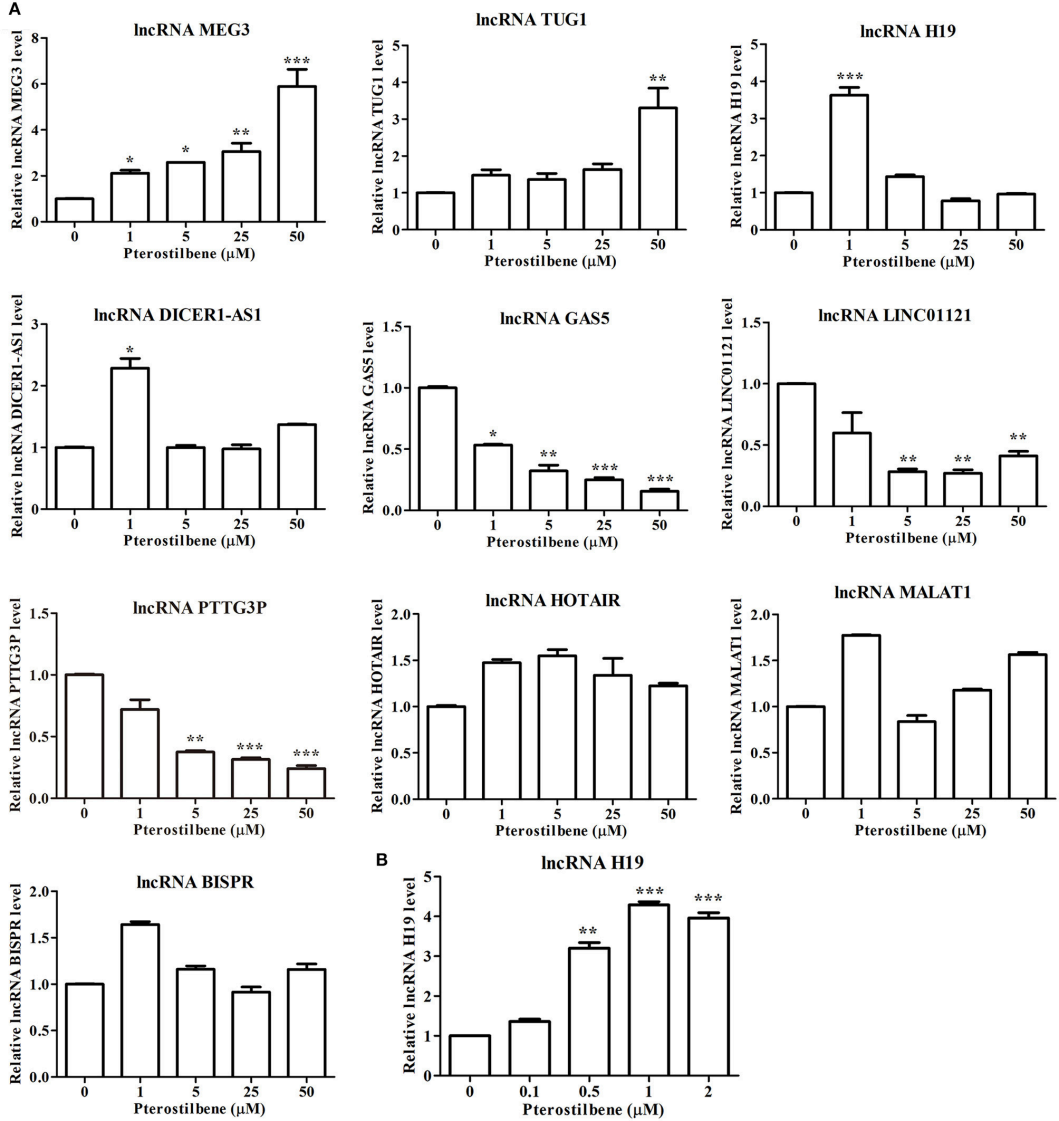
Pterostilbene influenced the expression of lncRNAs. **(A)** Cells were treated with pterostilbene at different concentrations (0, 1, 5, 25, and 50 μM) for 24 h with gene expression determined by qRT-PCR. **(B)** Expression of the lncRNA H19 in cells treated with 0, 0.1, 0.5, 1, and 2 μM pterostilbene for 24 h as determined by qRT-PCR. Reported values are mean ± SEM. *p < 0.05, **p < 0.01 and ***p < 0.001, indicate significant differences compared with the control group.

There is increasing evidence demonstrating that lncRNAs are critical for the initiation and progression of cancer. Therefore, to further uncover the underlying mechanisms of pterostilbene in cancer treatment, the present study attempted to examine candidate lncRNAs which have demonstrated regulation in cancer progression (Figure 11).

The expression of the lncRNA MEG3 was upregulated in MCF7 cells following pterostilbene treatment at various concentrations. There were no significant differences in the expression of TUG1 among the tested groups except for cells treated with 50 μM pterostilbene exhibiting upregulation. Interestingly, the expression of H19 and DICER1-AS1 increased in cells treated with 1 μM pterostilbene, but expression levels in cells treated with 5, 25, and 50 μM pterostilbene were similar to those of the control.

The expression of the lncRNA GAS5 decreased in cells treated with 1, 5, 25, and 50 μM pterostilbene, with LINC01121 and PTTG3P expression levels declining at concentrations of 5, 25, and 50 μM pterostilbene. Pterostilbene did not inhibit HOTAIR, MALAT1 or BISPR in MCF7 cancer cells.

### Sensitivity of Pterostilbene Inhibition of Cell Migration Was Enhanced via lncRNA H19 Knockdown

To obtain an insight into the underlying mechanisms of the involvement of lncRNAs in pterostilbene treatment, we focused on the role of H19. The expression of H19 was quantified in MCF7 cells after treatment with low concentrations of pterostilbene (0, 0.1, 0.5, 1, and 2 μM). The results of qPCR revealed that no significant differences in H19 expression were observed in the control compared with the 0.1 μM pterostilbene treatment, but expression increased in cells treated with 0.5, 1, and 2 μM pterostilbene.

**Figure 12:**
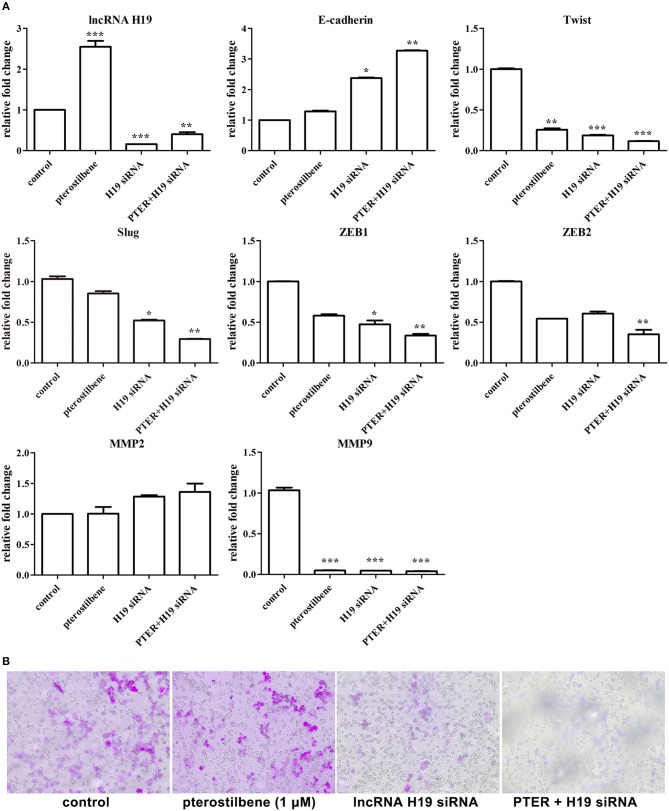
lncRNA H19 knockdown further impaired migration and EMT in cancer cells. **(A)** Gene expression in cells after pterostilbene (PTER) treatment and/or lncRNA H19 knockdown. Reported values are mean ± SEM. *p < 0.05, **p < 0.01 and ***p < 0.001, indicate significant differences compared with the control group. **(B)** Cell migration examined by Transwell assay.

Furthermore, the expression of lncRNA H19 was knocked down using specific siRNAs. As shown in Figure 12A, the expression of H19 exhibited significant knockdown through the use of an H19 siRNA pool. To emphasize the impact of lncRNA H19 knockdown on anti-tumor outcomes after pterostilbene treatment, a concentration of 1 μM was used. The results demonstrate that E-cadherin mRNA expression was significantly enhanced by 1 μM pterostilbene after H19 knockdown. Furthermore, the expression of Twist, Slug, Zeb1, and Zeb2 were further reduced by this combination. The Transwell assay was also performed to demonstrate any impact on cell migration. As shown in Figure 12B, both H19 knockdown alone and the combined treatment significantly inhibited the cell migration. Furthermore, the expression of the metastasis-related protein MMP9 decreased in the MCF7 cells treated with pterostilbene combined with H19 siRNAs (Figure 12A). However, MMP2 expression levels were similar in all tested groups.

## Discussion

The present study sought to elucidate the underlying mechanisms of anti-cancer effects exhibited by pterostilbene treatment in breast cancer cells. As expected, pterostilbene inhibited cell viability and induced apoptosis, autophagy, and ER stress in a dose- and time- dependent manner. In addition, the results demonstrated that such treatment promoted inhibition of the EMT process and regulated the expression of lncRNAs in breast cancer cells.

EMT is a complex cellular mechanism which performs an important role in cancer progression and metastasis, contributing to resistance to therapies. Increasing evidence suggests that novel therapies that block the induction of EMT would benefit patients with tumors undergoing metastasis (27). It is possible that phytochemical agents can inhibit EMT-inducing signals or downstream signal transduction pathways effectively. The effects of pterostilbene on EMT have been demonstrated previously in many studies (28). In agreement with these reports, suppression of E-cadherin expression and downregulation of a number of mesenchymal cell marker genes was observed in the pterostilbene-treated cells. Loss of E-cadherin expression is a hallmark of EMT and key to tumor progression (29). Therefore, increased E-cadherin expression observed in the pterostilbene treated cells indicates potential application of pterostilbene in cancer therapy. In addition, reduced phosphorylation of Akt is observed in pterostilbene-treated cells. Studies have shown that Akt activation accounts for decreased E-cadherin expression (30). It is possible that Akt signaling can act as a promising target for pterostilbene in the regulation of the EMT process. Phosphorylation of Erk (Thr202/Tyr204) also increased in the pterostilbene-treated cells. Activation of Erk and p38 MAPK signaling is associated with ovarian cancer tumorigenesis and metastasis (31). In fact, p38 MAPK, Akt, and Erk have been shown to have an association with EMT in tongue squamous carcinomas (32). Taken together, we demonstrated that pterostilbene-controlled cell migration and metastasis occur possibly via regulation of the crosstalk among these pathways.

The present study also investigated the potential mechanisms involved in lncRNA expression after pterostilbene treatment. As far as can be ascertained, few reports of the role of lncRNAs during pterostilbene treatment have been published. Its administration induced upregulation of many lncRNAs, including MEG3, TUG1, H19, and DICER1-AS1. Previous reports have indicated that enhanced expression of MEG3 renders colorectal cells more sensitive to oxaliplatin therapy by regulating the mir-141/PDCD4 axis (33). In human pituitary tumor-derived cells, enforced MEG3 expression was shown to significantly inhibit tumor growth (34). The expression of MEG3 increased even at a pterostilbene concentration of 1 μM, possibly indicating a key role for MEG3 in the anti-cancer properties of pterostilbene. Unexpectedly, many published reports suggest that the lncRNA TUG1 promotes cancer cell proliferation and invasion via different mechanisms in different cells (35, 36). The high concentrations of pterostilbene triggering upregulation of TUG1 suggest an anti-cancer role for TUG1 exists. Alternatively, high concentrations of pterostilbene would otherwise lead to chemotherapeutic resistance. The results of the present study also demonstrated decreased expression of the lncRNAs LINC01121, PTTG3P and HOTAIR following pterostilbene treatment. In pancreatic cancer, LINC01121 is shown to inhibit cell apoptosis and promote cell proliferation, migration and invasion via interaction of GLP1R in the control of the Camp/PKA signaling pathway (37). In hepatocellular carcinoma, enforced expression of the lncRNA PTTG3P facilitates cell proliferation, invasion, migration, and metastasis via activation of the PI3K/Akt pathway (38). These results suggest that pterostilbene possibly inhibits cell proliferation and EMT via a decrease in LINC01121 and PTTG3P expression. Of the lncRNAs that exhibit fluctuating expression after pterostilbene treatment, H19, and DICER1-AS1 are the most interesting in that their upregulation occurred at a pterostilbene treatment concentration of 1 μM. DICER1-AS1 has been shown to enhance proliferation, invasion and autophagy through the miR-30b/ATG5 axis in osteosarcoma cells (39). However, studies of DICER1-AS1 are, so far, limited in number.

To gain an insight into the underlying mechanisms, the present study focused further on the function of the lncRNA H19 in cells after treatment with pterostilbene. Whether H19 is oncogenic or tumor-suppressive remains controversial, but the majority of reports suggest it provides an oncogenic function in many cancers (40). In addition, mounting evidence suggests that H19 promotes EMT (19, 41, 42). Upregulation of H19 at only low concentrations of pterostilbene is possibly due to the different competing endogenous RNA (ceRNA) mechanisms involved with H19. H19 has been shown to mediate both EMT and MET plasticity by the differential sponging of miR-200b/c and let-7b in breast cancer cells (19). The promotion of H19 expression may possibly establish chemoresistance to pterostilbene in MCF7 cells. Many previous studies have suggested that silencing H19 expression could overcome chemoresistance. In colorectal cancer, it has been shown that H19 becomes significantly upregulated in methotrexate-resistant cells, with H19 knockdown sensitizing methotrexate resistance in this cell line (43). In triple negative breast cancer, H19 expression is significantly higher in paclitaxel-resistant cells than in paclitaxel-sensitive cells, with downregulation of H19 restoring chemo-sensitivity in the resistant cells after mediation of the Akt signaling pathway (44). In human glioma, the expression of H19 has been shown to also significantly increase in temozolomide-resistant cells, with silencing of H19 decreasing resistance to temozolomide by inhibition of EMT (45). Therefore, knockdown of the expression of the lncRNA H19 via siRNAs in this study aimed to determine the impact of H19 on the EMT process. The results confirm that cellular migration was further decreased by pterostilbene when combined with H19 knockdown, indicating that sensitivity to pterostilbene was enhanced by a decrease in the abundance of H19.

In conclusion, the present study demonstrated that pterostilbene induced apoptois, suppressed EMT and modified the expression of lncRNAs in breast cancer cells. In addition, knockdown of the lncRNA H19 enhanced the inhibition of EMT by treatment with pterostilbene. The results of our study indicate that combining pterostilbene treatment with lncRNA interference present an alternative strategy for cancer therapy.

## Author Contributions

YoH, HO, and YuH conceived and designed the experiments. YoH, JD, YM, TL, and YG performed the experiments and analyzed the data. YoH wrote the manuscript. HO and YuH reviewed the manuscript. All authors approved the manuscript.

### Conflict of Interest Statement

The authors declare that the research was conducted in the absence of any commercial or financial relationships that could be construed as a potential conflict of interest.
